# Double Trouble: Unveiling the Rare Co-occurrence of Neurofibromatosis Type 1 and Multiple Sclerosis

**DOI:** 10.7759/cureus.90034

**Published:** 2025-08-13

**Authors:** Simranpreet Singh, Mukesh Kumar, Shivangi, Joseph J Melvin, Om Parkash

**Affiliations:** 1 Neurology, Tower Health Medical Group, West Reading, USA; 2 Child Neurology, St. Christopher's Hospital for Children, Philadelphia, USA; 3 Internal Medicine, Tower Health Medical Group, West Reading, USA

**Keywords:** cns demyelinating disease, dawson's fingers, multiple sclerosis phenotypes, neurofibromatosis type 1 (nf1), ocrelizumab

## Abstract

Neurofibromatosis type 1 (NF1) is a genetic disorder, whereas multiple sclerosis (MS) is an autoimmune, demyelinating disease of the central nervous system (CNS). The co-occurrence of both pathologies is rare, and overlapping symptoms can pose diagnostic challenges.​ We present a rare case of coexisting NF1 and multiple sclerosis (MS) in a patient with a known history of NF1.

An 18-year-old female patient with known NF1 (genetically diagnosed) presented to the hospital for new-onset falls for one week and blurry vision for two weeks. The examination revealed increased tone in bilateral lower extremities​ and brisk knee reflexes, slightly wide-based gait, positive Romberg's test, and dysmetria on finger-to-nose testing on the left. Chart review revealed that the patient experienced hearing loss six months ago. A magnetic resonance imaging (MRI) of the brain performed at that time demonstrated focal areas of signal intensity (FASI) consistent with stigmata of neurofibromatosis type 1 (NF1), without evidence of a cerebellopontine angle mass such as a vestibular schwannoma. The usual decrease in FASI lesions was not seen, raising suspicion of a demyelinating etiology. Repeat MRI of the brain with contrast on admission revealed progression of multiple centrally enhancing white matter lesions, Dawson's fingers (ovoid, finger-like, hyperintense lesions oriented perpendicular to the lateral ventricles), and bilateral optic nerve enhancement. MRI of the spine showed multiple T2 hyperintensities throughout the spine. The appearance of these intracranial and intraspinal lesions were consistent with MS. Cerebrospinal fluid (CSF) studies showed positive oligoclonal bands and elevated immunoglobulin G (IgG) index. She received five days of high-dose intravenous methylprednisolone, leading to significant improvement in her gait, and was started on ocrelizumab infusions on post-hospital discharge follow-up.

This case underscores the importance of recognizing the potential coexistence of NF1 and MS, especially in patients presenting with new neurological symptoms not fully explained by one disorder. Sudden sensorineural hearing loss, defined as a decrease of 30 dB or greater, affecting at least three consecutive audiometric frequencies, occurring within a few hours up to three days, affects 4%-10% of MS patients between relapses or remission and is common in NF2. Early neuroimaging, careful interpretation of lesion characteristics, and CSF analysis are essential to establish the diagnosis and initiate prompt treatment. Given the overlapping radiological and clinical features, NF1 may mask demyelinating pathology, delaying diagnosis and treatment. Awareness of this rare association may facilitate earlier recognition, especially when imaging or symptoms deviate from classical NF1 progression. This case also highlights the evolving understanding of central nervous system (CNS) immune interactions in neurogenetic syndromes. Future research is warranted to determine whether patients with NF1 harbor an intrinsic predisposition to autoimmune CNS disorders such as MS, and whether neurofibromin deficiency plays a role in immune dysregulation. Ultimately, this case calls for a high index of clinical suspicion, multidisciplinary collaboration, and judicious use of advanced diagnostics in managing patients with complex neurological disorders.

## Introduction

Neurofibromatosis type 1 (NF1), also known as von Recklinghausen's disease, is an autosomal dominant neurocutaneous disorder caused by mutations in the *NF1* gene located on chromosome 17q11.2. This gene encodes neurofibromin, a tumor suppressor protein involved in the Ras-mitogen-activated protein kinase (Ras/MAPK) pathway. NF1 is known for its classical cutaneous manifestations, neurofibromas, café au lait macules, axillary freckling, and Lisch nodules. However, central nervous system (CNS) involvement is also common and includes optic pathway gliomas, scoliosis, and focal areas of signal intensity in the brain, often mistaken for white matter lesions. In contrast, multiple sclerosis (MS) is a chronic, autoimmune, demyelinating disorder of the CNS that typically presents in young adults. It is characterized by lesions disseminated in time (occurring at different points in time) and space (distinct areas of the central nervous system, often involving the periventricular, juxtacortical, infratentorial, and spinal cord white matter). Clinical manifestations include motor and sensory disturbances, visual symptoms, cerebellar dysfunction, and cognitive impairment [1,2]. The co-occurrence of NF1 and MS is exceedingly rare, with only a handful of reported cases in the literature [3-5]. The mechanisms underpinning their association remain speculative, with hypotheses ranging from shared genetic susceptibilities to environmental triggers, altered immune surveillance, and overlapping CNS microenvironments. Because of shared clinical and radiological features, the presence of NF1 can obscure or delay the diagnosis of MS, particularly in the early stages [6-8]. This report aims to present a rare case of coexisting NF1 and MS in a young adult, highlighting the diagnostic challenges that arise due to overlapping neurological symptoms, imaging findings, and disease progression. Through the presentation of this clinical vignette, we explore the pathophysiological theories, clinical implications, and the importance of maintaining a high index of suspicion for dual pathology in complex neurogenetic patients.

## Case presentation

An 18-year-old female patient with a known diagnosis of genetically confirmed neurofibromatosis type 1 (NF1) presented to the emergency department with acute-onset neurological symptoms. She reported recurrent falls over the preceding week, accompanied by progressively worsening blurry vision in both eyes for the past two weeks. There was no associated history of trauma, fever, upper respiratory tract infection, or constitutional symptoms such as weight loss or fatigue.

Her past medical history included a mild learning disability, multiple cutaneous neurofibromas, and documented hearing loss approximately six months ago. There was no family history of demyelinating diseases or other autoimmune disorders. Her vitamin D levels were within the normal range, and she had no history of either active or passive smoking.

On neurological examination, the patient demonstrated hypertonia in bilateral lower extremities, with brisk patellar reflexes and sustained ankle clonus. Her gait was wide-based and unsteady, with a positive Romberg sign, suggestive of proprioceptive or cerebellar involvement. Cerebellar examination revealed dysmetria on the left side during the finger-to-nose test. Visual acuity testing showed mild bilateral reduction; however, fundoscopic examination was unremarkable, with no signs of papilledema or optic disc pallor. Cranial nerve examination was intact, and no sensory level or dermatomal deficits were identified.

Cutaneous examination revealed numerous café au lait macules and axillary freckling, findings consistent with the established diagnosis of NF1. There were no signs of neurocutaneous stigmata or skin lesions concerning for malignancy.

A review of prior neuroimaging performed six months earlier, during her evaluation for sensorineural hearing loss, showed multiple areas of T2-weighted hyperintensities in the basal ganglia and brainstem. These lesions were consistent with focal areas of signal intensity (FASI), a hallmark of NF1. Typically, FASI lesions are non-enhancing and tend to regress over time. However, the patient's imaging had shown no reduction in lesion burden, and some areas exhibited subtle atypical features.

**Figure 1:**
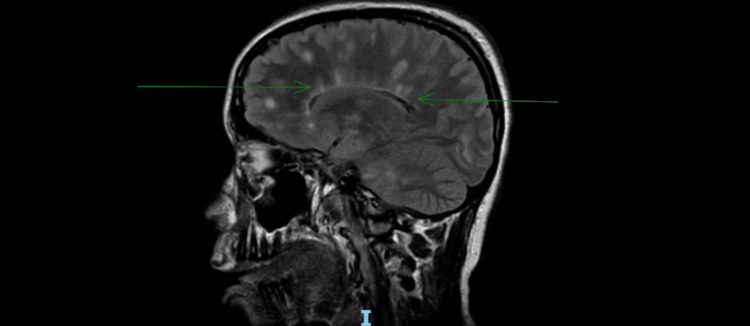
MRI of the brain T2 fluid-attenuated inversion recovery sequence (sagittal view) showing Dawson's fingers; also, above the Dawson's fingers on the right are typical FASI lesions MRI: magnetic resonance imaging, FASI: focal areas of signal intensity

Given her evolving neurological symptoms and absence of expected radiological resolution, differentials considered were MS, acute disseminated encephalomyelitis (ADEM), neuromyelitis optica spectrum disorder (NMOSD), and myelin oligodendrocyte glycoprotein-associated disease (MOGAD), for which further investigation was pursued. A repeat magnetic resonance imaging (MRI) of the brain with contrast revealed multiple new enhancing lesions within the periventricular and juxtacortical white matter. Several periventricular lesions demonstrated classic "Dawson's fingers" appearance, highly suggestive of MS (Figure 1).

**Figure 2:**
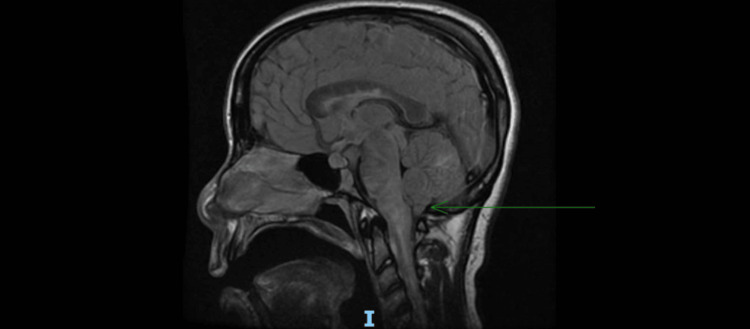
MRI of the brain T2 fluid-attenuated inversion recovery sequence (sagittal view) showing swelling of the brainstem and cerebellum with effacement of ventricles MRI: magnetic resonance imaging

Additionally, there was enhancement of bilateral optic nerves and notable swelling in the brainstem and cerebellum with effacement of the ventricle (Figure 2).

**Figure 3:**
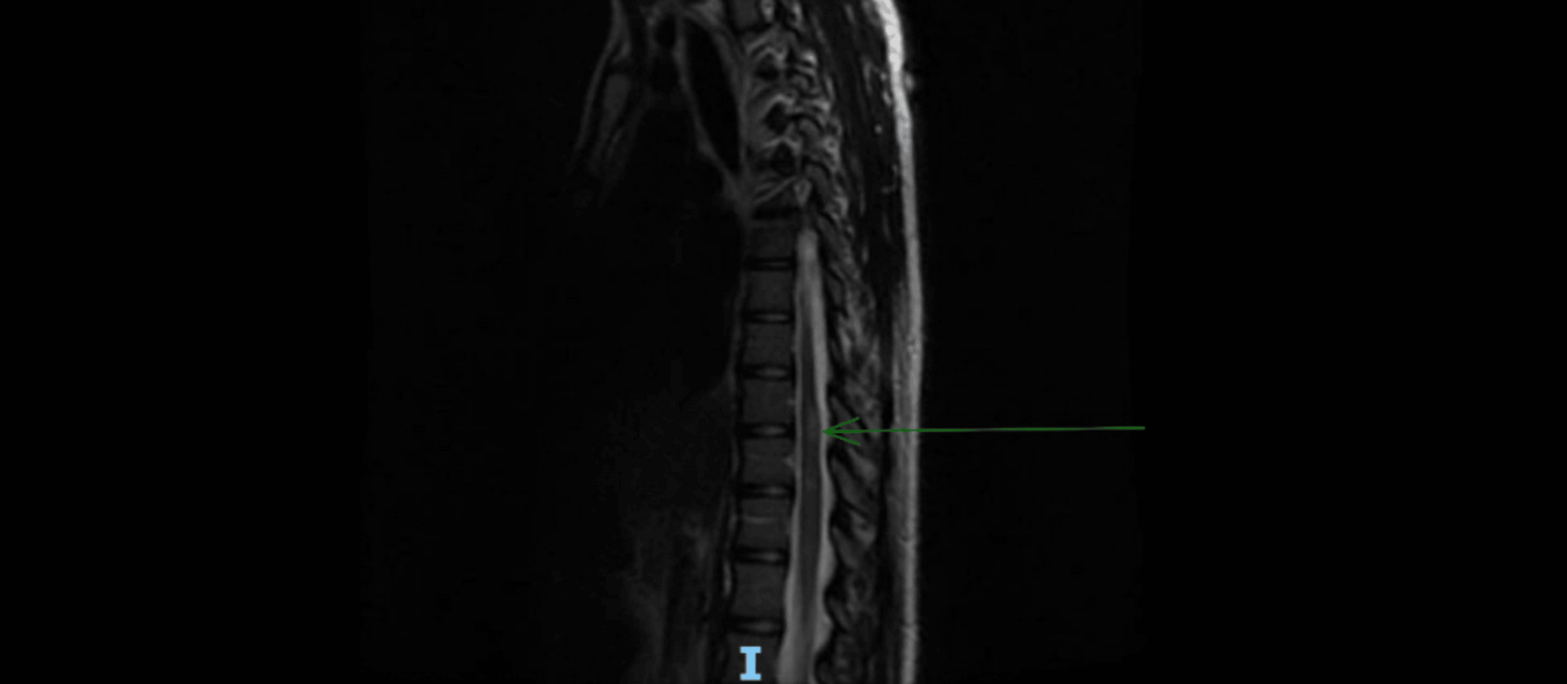
MRI of the thoracic spine T2 fast-recovery fast spin-echo sequence (sagittal view) showing T10-T12 hyperintensities MRI: magnetic resonance imaging

MRI of the cervical and thoracic spine with contrast revealed patchy T2 hyperintensities without associated cord expansion or enhancement (Figure 3).

The distribution and appearance of these lesions raised further concern for an underlying demyelinating process.

A lumbar puncture (LP) was subsequently performed. The cerebrospinal fluid (CSF) analysis (Table 1) shows normal color and appearance with mildly elevated lymphocytes (90%), normal protein and glucose levels, and the presence of four oligoclonal bands that were absent in serum, consistent with intrathecal immunoglobulin G (IgG) synthesis. These findings fulfilled the diagnostic criteria for MS, and other differentials were excluded based on imaging characteristics, negative antibody testing, and clinical evolution. Additional investigations, including infectious, paraneoplastic, and metabolic panels, returned unremarkable, ruling out alternative etiologies.

**Table 1:** Cerebrospinal fluid studies Bold values: elevated levels CSF: cerebrospinal fluid, PCR: polymerase chain reaction, NMO: neuromyelitis optica, AQP4: aquaporin-4, MOG: myelin oligodendrocyte glycoprotein, ACE: angiotensin-converting enzyme

Component	Result	Reference range
Color	Colorless	NA
Appearance	Clear	NA
Total nucleated cells	10/µL	0-5/µL
Red blood cells	<1,000/µL	0/µL
Cell differential	Lymphocytes: 90%, macrophages: 10%	Lymphocytes: 40%-80%, macrophages: 15%-45%
Protein	35 mg/dL	15-45 mg/dL
Glucose	52 mg/dL	40-75 mg/dL
Oligoclonal bands	4 unique bands in CSF, not present in serum	NA
CSF immunoglobulin G	7.5 mg/dL	0.0-6.7 mg/dL
CSF albumin	22 mg/dL	7-29 mg/dL
CSF IgG/albumin ratio	0.34	0.00-0.25
CSF IgG index	1.3	0.0-0.7
JC virus PCR	Not detected	NA
Paraneoplastic panel	Negative	NA
NMO (AQP4) antibodies	Negative	NA
MOG antibodies	Negative	NA
Mayo ENC2 panel	Negative	NA
ACE	<1.5 U/L	0.0-2.5 U/L

Based on the 2017 revised McDonald criteria for MS, the patient was diagnosed with relapsing-remitting multiple sclerosis on day 2 after MRI and LP results. She was treated with high-dose intravenous methylprednisolone on day 2 (1 g daily for five days) during her 7-day hospital course, which led to substantial improvement in her neurological symptoms. Her gait and visual disturbances improved significantly.

She was discharged home in stable condition with plans for close outpatient follow-up with neurology and neuro-ophthalmology. Her vision had fully recovered by the time of discharge. Twenty days after discharge, once clinically stable, she began treatment with ocrelizumab, a B-cell-depleting disease-modifying therapy for long-term MS management, starting with two loading doses 15 days apart, followed by maintenance infusions every six months. After discharge, she was able to walk a block using a walker and continued participating in physical therapy. She now no longer requires the walker, has not reported significant fatigue, and has experienced no falls, stumbling, or shuffling, and can walk with a steady gait.

## Discussion

The co-occurrence of NF1 and MS is exceedingly rare, with only a limited number of cases reported in the literature. NF1, caused by mutations in the *NF1* gene on chromosome 17, is a neurocutaneous disorder characterized by benign tumor formation, skin manifestations, and focal areas of signal intensity (FASI) on MRI [1,2]. In contrast, MS is an autoimmune demyelinating disease of the central nervous system, with hallmark imaging findings such as periventricular hyperintensities, Dawson's fingers, and spinal cord lesions. The overlapping clinical and radiological features of these two diseases present a significant diagnostic challenge.

Our 18-year-old female patient with a known diagnosis of NF1 presented with neurological symptoms, including falls, visual disturbance, gait imbalance, and sensorineural hearing loss. While these features may be attributed to NF1, the atypical progression of lesions on imaging, combined with new enhancing demyelinating lesions and corroborative CSF findings (positive oligoclonal bands and elevated IgG index), led to a definitive diagnosis of MS.

Several hypotheses exist regarding the pathophysiological overlap of NF1 and MS [3-5]. It has been suggested that mutations in the *NF1* gene might influence immune regulation or glial cell function, thereby increasing susceptibility to demyelinating diseases. Additionally, the presence of FASI in NF1 may obscure early MS lesions, delaying diagnosis. In our case, the initial MRI findings were attributed to NF1, and it was only the progression of symptoms and evolution of imaging that prompted reconsideration and identification of coexisting MS.

The case also highlights that sensorineural hearing loss, commonly associated with both NF1 (particularly when vestibular schwannomas are present, although more common in NF2) and MS (due to demyelination of the auditory pathway), can be a shared early symptom. A high index of suspicion, especially in the presence of atypical MRI evolution or new symptoms, is crucial for timely recognition and treatment of MS in patients with NF1.

## Conclusions

This case underscores the importance of recognizing the potential coexistence of NF1 and MS, especially in patients presenting with new neurological symptoms not fully explained by one disorder. Early neuroimaging, careful interpretation of lesion characteristics, and CSF analysis are essential to establish the diagnosis and initiate prompt treatment. Given the overlapping radiological and clinical features, NF1 may mask demyelinating pathology, delaying diagnosis and treatment. Awareness of this rare association may facilitate earlier recognition, especially when imaging or symptoms deviate from classical NF1 progression. This case also highlights the evolving understanding of CNS immune interactions in neurogenetic syndromes. Future research is warranted to determine whether patients with NF1 harbor an intrinsic predisposition to autoimmune CNS disorders such as MS, and whether neurofibromin deficiency plays a role in immune dysregulation. Ultimately, this case calls for a high index of clinical suspicion, multidisciplinary collaboration, and judicious use of advanced diagnostics in managing patients with complex neurological disorders.
